# ROS-talk – how the apoplast, the chloroplast, and the nucleus get the message through

**DOI:** 10.3389/fpls.2012.00292

**Published:** 2012-12-27

**Authors:** Alexey Shapiguzov, Julia P. Vainonen, Michael Wrzaczek, Jaakko Kangasjärvi

**Affiliations:** Division of Plant Biology, Department of Biosciences, University of HelsinkiHelsinki, Finland

**Keywords:** ROS signaling, apoplast, chloroplasts, retrograde signaling, *Arabidopsis thaliana*

## Abstract

The production of reactive oxygen species (ROS) in different plant subcellular compartments is the hallmark of the response to many stress stimuli and developmental cues. The past two decades have seen a transition from regarding ROS as exclusively cytotoxic agents to being considered as reactive compounds which participate in elaborate signaling networks connecting various aspects of plant life. We have now arrived at a stage where it has become increasingly difficult to disregard the communication between different types and pools of ROS. Production of ROS in the extracellular space, the apoplast, can influence their generation in the chloroplast and both can regulate nuclear gene expression. In spite of existing information on these signaling events, we can still barely grasp the mechanisms of ROS signaling and communication between the organelles. In this review, we summarize evidence that supports the mutual influence of extracellular and chloroplastic ROS production on nuclear gene regulation and how this interaction might occur. We also reflect on how, and *via* which routes signals might reach the nucleus where they are ultimately integrated for transcriptional reprogramming. New ideas and approaches will be needed in the future to address the pressing questions of how ROS as signaling molecules can participate in the coordination of stress adaptation and development and how they are involved in the chatter of the organelles.

## INTRODUCTION

During their life plants face a vast set of environmental challenges: extreme changes in ambient illumination, temperature, and humidity, differences in soil salinity, attack by pathogens and herbivores, mechanical wounding, etc. To withstand all these challenges, plants have developed a repertoire of signaling pathways that is unparalleled in its complexity among living organisms. Signaling through plant hormones (Vanstraelen and Benková, 2012), cell surface receptors (Geldner and Robatzek, 2008), and light perception by plastids and photoreceptors (Kami et al., 2010) are integrated in the cell to eventually reprogram gene expression and metabolism and shape strategic decisions on plant stress response and development (Jaillais and Chory, 2010).

**FIGURE 1:**
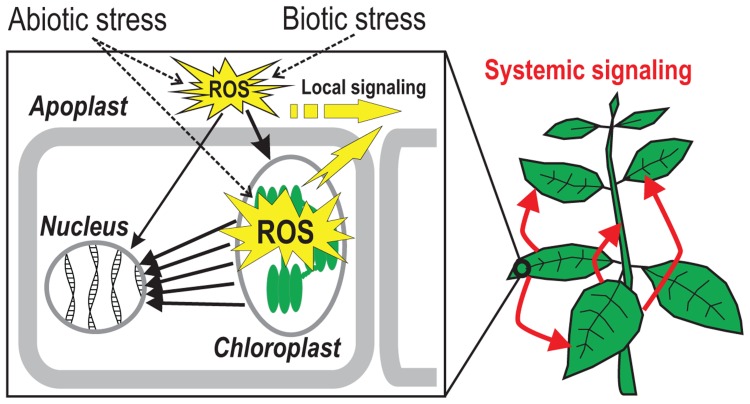
**Reactive oxygen species (ROS)-talk in plant cells**. Biotic and abiotic stimuli lead to the generation of ROS in the apoplast which is subsequently communicated to the inside of the cell where the signal leads to an increase in chloroplastic ROS production. The chloroplast can further amplify the signal and transmit it to the nucleus *via *various cytosolic signaling networks. Apoplastic ROS signaling can also reach the nucleus through cytosolic pathways directly. Yellow arrows demonstrate intracellular transmission of apoplastic and chloroplastic ROS-induced signals where they connect neighboring cells (local signaling) or participate in long-distance (“systemic”) signaling throughout the plant (red arrows).

A critical role in this signal integration and decision-making is played by a class of reactive forms of the molecular oxygen, collectively referred to as reactive oxygen species (ROS; Murphy et al., 2011; Kangasjärvi et al., 2012). ROS, including singlet oxygen (^1^O_2_), superoxide (${\text{O}}_{2}^{•-}$), hydrogen peroxide (H_2_O_2_), and hydroxyl radical (^•^OH) are unavoidable by-products of aerobic metabolism (Imlay, 2003, 2008; Ogilby, 2010) which have traditionally been regarded mainly as damaging cytotoxic agents. In line with this view, life has developed a plethora of ROS scavenging systems including the low-molecular weight compounds ascorbic acid and glutathione (Foyer and Noctor, 2011) as well as different classes of antioxidant enzymes (Apel and Hirt, 2004). During the recent years, however, a new concept has emerged where ROS play important signaling roles during development and stress responses, and controlled production of ROS acts as a signal. ROS are generated in many compartments of plant cells. Whereas the “ROS landscape” of the animal cell is dominated by mitochondria as the main source of ROS (Marchi et al., 2012), the role of these organelles in ROS production in plants is more subtle (Dutilleul et al., 2003; Suzuki et al., 2012) and is not addressed in this review. Apart from mitochondria, ROS are produced in the chloroplasts, the peroxisomes, and the apoplast, as well as in less commonly known locations, the nucleus and the endoplasmic reticulum (Overmyer et al., 2003; Ashtamker et al., 2007; Foyer and Noctor, 2009; Jaspers and Kangasjärvi, 2010; Mazars et al., 2010). Yet uncharacterized signaling networks between the organelles that employ ROS as second messengers have recently raised considerable interest (**Figure 1**). For example, ROS that are produced in the chloroplast have been implicated as intermediates in retrograde signaling from chloroplast to nucleus during acclimation of photosynthesis (Nott et al., 2006; Galvez-Valdivieso and Mullineaux, 2010). Intriguingly, however, it has recently been realized that the role of this signaling goes beyond optimization of photosynthesis: chloroplastic ROS production and photosynthetic functions are also regulated by cues that are perceived in the cell wall, frequently referred to as the extracellular space or the apoplast (Padmanabhan and Dinesh-Kumar, 2010). Thus, the *sensu stricto *retrograde signaling (from chloroplast to nucleus) can also be regarded as a part of a larger network where apoplastic signals induce the generation of ROS in the chloroplast, which in turn leads to regulation of nuclear gene expression by several still uncharacterized, but at least partially chloroplast-derived, ROS-dependent retrograde signals.

## ROS IN THE APOPLAST

**FIGURE 2:**
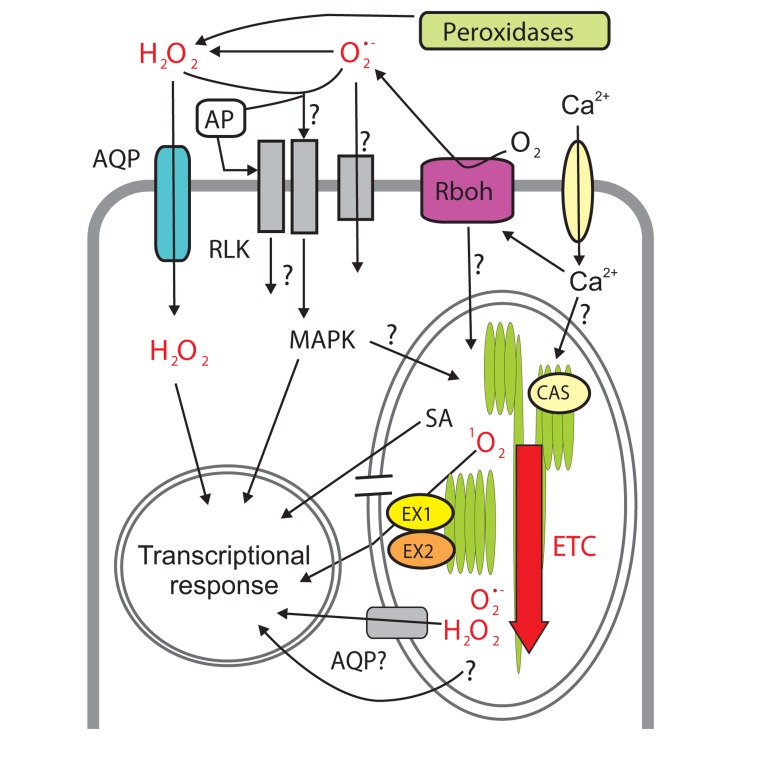
**Reactive oxygen species (ROS) signaling networks connecting apoplast, chloroplast and nucleus**. Apoplastic ROS are produced by extracellular peroxidases (hydrogen peroxide; H_2_O_2_) and plasma membrane-bound NADPH oxidases, Rboh. Superoxide (${\text{O}}_{2}^{•-}$) is then converted to H_2_O_2_. H_2_O_2_ (and possibly ${\text{O}}_{2}^{•-}$) might enter the cell through plasma membrane channels (aquaporins, AQP) and/or react with extracellular (apoplastic protein, AP) or transmembrane sensor proteins (e.g., receptor-like kinases, RLKs) ultimately resulting in changes in gene expression through intracellular signaling pathways, involving, for example, MAPKs (mitogen-activated protein kinases). Extracellular ROS production is sensed *via* yet unknown mechanisms in the chloroplast where ROS generation by the electron transfer chain (ETC) subsequently increases. Singlet oxygen (^1^O_2_) and ${\text{O}}_{2}^{•-}$/H_2_O_2__2_O here should be changed to H_2_O_2_. are produced in different domains of ETC. Elevated ROS inside the chloroplast results in transcriptional reprogramming through identified (e.g., EXECUTER1/2, EX1/EX2, rupture of chloroplast envelope) and unknown components of the retrograde signaling but also through hormone signaling, e.g., increased production of the stress hormone salicylic acid (SA). Channel proteins (AQP) might also allow ROS leak from the chloroplast to the cytoplasm. Calcium (Ca^2+^) is involved in the regulation of ROS production in the apoplast and the chloroplast. In the latter case it acts through the sensory protein CALCIUM-SENSING RECEPTOR (CAS) but the mechanisms are still unclear.

Likely candidates involved in the apoplast-to-chloroplast signaling are ROS produced in the cell wall. Their accumulation in response to different abiotic and biotic stimuli during the so-called apoplastic “oxidative burst” depends on several classes of enzymes, including cell wall peroxidases (Bindschedler et al., 2006) and plasma membrane NADPH oxidases (**Figure 2**; Torres et al., 2002; Suzuki et al., 2011). The latter enzymes, commonly known as respiratory burst oxidase homologs (Rboh) are transmembrane flavoproteins that oxidize cytoplasmic NADPH, translocate electrons across plasma membrane and reduce extracellular ambient (triplet) oxygen to yield ${\text{O}}_{2}^{•-}$ in the cell wall. Due to its charge, this short-lived ROS is unable to passively cross the lipid bilayer and remains in the apoplast, where it is rapidly converted into another species, H_2_O_2_, either spontaneously or in a reaction catalyzed by the superoxide dismutase (SOD; Browning et al., 2012). The functions of plant NADPH oxidases stretch beyond stress responses and include roles in development (Sagi and Fluhr, 2006; Takeda et al., 2008), in sodium transport in the xylem sap (Jiang et al., 2012), and intriguingly also in long-distance (“systemic”) ROS signaling (Miller et al., 2009). In Arabidopsis wounding, heat stress, high light, and increased salinity result in RbohD-dependent systemic spread of the oxidative burst along the rosette leaves. The signal is triggered by intracellular Ca^2+^ spiking at the wounding site. It is propelled by accumulation of ROS in the apoplast and by – still unidentified – symplastic signals, one of which might be ROS production in chloroplasts: results by Joo et al. (2005) suggest that chloroplastic ROS is required for intercellular ROS signaling. This ROS “wave” travels across an Arabidopsis rosette at a rate of approximately 8 cm per minute (Miller et al., 2009). Taken together, the currently available data suggests different roles for ROS in strictly localized signaling events but also in systemic signaling.

We have obtained a good understanding of the processes in which apoplastic ROS are involved, but how they are perceived by plant cells remains unclear. It is not known how the signal is transmitted to the cytoplasm, the chloroplasts and eventually the nucleus and what are the interactions between the different subcellular compartments. The possibility of ${\text{O}}_{2}^{•-}$ itself being the mediator of downstream signaling would require superoxide-specific extracellular receptors or anion channels in the direct vicinity to the site of ${\text{O}}_{2}^{•-}$ production (Browning et al., 2012). Anion channels have been shown to mediate superoxide import in mammalian cells (Hawkins et al., 2007) thereby linking extracellular and intracellular ROS signaling. Analogous systems in plants have so far not been identified. Unlike superoxide, the H_2_O_2_ molecule is relatively stable (with a half-life of ~1 ms) under physiological conditions and in many respects resembles a molecule of water. Its dipole moment, similar to that of H_2_O, limits passive diffusion of H_2_O_2_ through biological membranes. Possible candidates for the import of apoplastic H_2_O_2_ are aquaporins (**Figure 2**), a ubiquitous family of channel proteins that has undergone an extensive expansion in vascular plants (Zardoya, 2005; Soto et al., 2012). Recent studies have identified several aquaporins as specific H_2_O_2_ transporters in Arabidopsis (Bienert et al., 2007; Dynowski et al., 2008; Hooijmaijers et al., 2012). However, further research is required to assess the role of H_2_O_2_ transport during the oxidative burst. In addition to transport across membranes, ${\text{O}}_{2}^{•-}$ and H_2_O_2_ may be sensed by a number of apoplastic compounds. Oxidation of extracellular pools of glutathione and ascorbic acid might play a role in transmitting the redox signal to the cytosol (Destro et al., 2011; Foyer and Noctor, 2011; Noctor et al., 2012). ROS can also be perceived by the apoplastic proteins and/or plasma membrane-localized receptors through redox modification of their cysteine residues (**Figure 2**). Those putative receptors or other sensory systems for extracellular ROS in plants have so far remained elusive, but for example, several classes of receptor-like protein kinases (RLKs) with cysteine-rich extracellular domains (most notably the CYSTEINE-RICH RLKs, CRKs) have been suggested to be involved in ROS perception (Shiu and Bleecker, 2003; Wrzaczek et al., 2010).

## FROM BEYOND TO HERE, SIGNALS FROM THE EXTRACELLULAR SPACE

What happens in the plant cell after an extracellular oxidative burst has been triggered? A connection of apoplastic and chloroplastic ROS into common signaling networks during the plant stress response is evident in various model systems and processes (Joo et al., 2005; Vahisalu et al., 2010), although it is mechanistically still largely unexplained. The results suggest that the apoplastic ROS signal is transduced to the chloroplasts, where a secondary ROS production is initiated. This signal transmission might use cytosolic signaling components. Also, the location of chloroplasts close to the plasma membrane might facilitate direct communication between the two organelles. Thus, the chloroplast can act as an “amplifier,” or “execute” the signal received from the apoplast (**Figure 1**).

One of the examples of such a role of chloroplasts is the plant immune response to pathogens that is accompanied by a bi-phasic accumulation of ROS. The first phase occurs within tens of minutes from the onset of infection. It is mostly apoplastic and is tightly linked to NADPH oxidase activity (**Figure 2**). The second increase in ROS production happens several hours after the pathogen attack (Lamb and Dixon, 1997; Jones and Dangl, 2006). During this stage of response the infected cells might undergo programmed cell death (PCD) leading to the collapse of the infected tissue and, in the case of biotrophic pathogens, to suppression of pathogen growth. This specialized form of pathogen-triggered PCD is a part of the hypersensitive response (HR). Different subcellular compartments including apoplast, chloroplasts, mitochondria, and peroxisomes contribute to ROS production during HR, but a growing amount of evidence suggests a crucial role for the chloroplast in this process (Yao and Greenberg, 2006; Liu et al., 2007; Zurbriggen et al., 2009, 2010). Silencing of the chloroplast redox proteins peroxiredoxin and NADPH-dependent thioredoxin reductase C that scavenge chloroplastic H_2_O_2_ led to spreading PCD in response to application of coronatine, a phytotoxin with structural similarity to jasmonic acid produced by several pathogenic strains of *Pseudomonas *(Ishiga et al., 2012). The involvement of chloroplasts in plant immunity is further supported by the observation that the pathogen resistance of plants differs between light and dark (Roden and Ingle, 2009) and by the fact that several bacterial and viral elicitors interact with chloroplast-targeted proteins or are imported into chloroplasts (Padmanabhan and Dinesh-Kumar, 2010). Thus, not only the apoplast and the cytosol, but also the chloroplasts are strategic battlefields during the defense against pathogens (Padmanabhan and Dinesh-Kumar, 2010).

Chloroplast-generated ROS are not only involved in initiating and promoting cell death during the HR, but also in the up-regulation of defense-related genes, down-regulation of photosynthesis genes and even in limiting the spread of the cell death (Straus et al., 2010). For example, a significant portion of the genes induced by artificial metabolic overproduction of H_2_O_2_ *via* expression of glyoxylate oxidase in the chloroplasts (Balazadeh et al., 2012), are also induced by chitin, a well-known elicitor of the apoplastic oxidative burst and downstream pathogen defense responses. Similarly, silencing of thylakoid ascorbate peroxidase (tAPX) led to an increase in H_2_O_2_ production and to activation of defense responses (Maruta et al., 2012), including the accumulation of the stress hormone salicylic acid (SA), a central mediator of plant pathogen defense. These examples underline that H_2_O_2_ accumulation in the chloroplast and the related retrograde signaling are involved in the activation of defense genes during responses to pathogens.

## CHLOROPLASTS AS THE PET PEEVE OF THE PLANT CELL

Why does the plant cell involve the chloroplast, the major site of energy production and biosynthesis in stress responses? One explanation is that photosynthesizing chloroplasts continuously produce ROS due to numerous electron transfer reactions in the presence of oxygen (Foyer and Noctor, 2003; Asada, 2006). Hence, in the photosynthesizing plant tissues chloroplasts are able to produce the most massive pools of ROS among different subcellular compartments.

Generation of ROS in the chloroplasts depends on multiple aspects of chloroplast physiology including photosynthesis, gene expression, chlorophyll (tetrapyrrole) biosynthesis, and hormonal control (Asada, 2006; Sierla et al., 2012). For example, the production of ${\text{O}}_{2}^{•-}$ /H_2_O_2_ by photosystem I (PS I) varies according to changing photosynthetic electron transfer and CO_2_ fixation rate. Extracellular stimuli, such as recognition of bacterial components by the plasma membrane receptors, can rapidly regulate chloroplastic functions. During plant–pathogen interactions the cues perceived in the apoplast trigger MAPK cascades (**Figure 2**) and result in fast down-regulation of photosynthetic genes and accumulation of H_2_O_2_ in chloroplasts that is necessary for initiation of HR-mediated cell death (Liu et al., 2007). Another example is the transient decrease in the ability of PS II to dissipate excessive light energy as heat *via* non-photochemical quenching (NPQ) at an early stage of pathogen recognition. This decrease in NPQ makes chloroplasts more predisposed to the production of ROS, which might be a priming mechanism for chloroplast ROS signaling at later stages of immune response (Göhre et al., 2012). Several chloroplastic redox hubs, including the plastoquinone as well as the glutathione pools and the thioredoxin system, provide not only dynamic local regulation of photosynthesis, but also might communicate the chloroplast redox status to the cytosol (Marty et al., 2009; Bashandy et al., 2010; Foyer and Noctor, 2011; Noctor et al., 2012; Rochaix, 2012). For example, the redox state of plastoquinone, a component of photosynthetic electron transfer chain, is monitored through the thylakoid-associated protein kinase STATE TRANSITION 7 (STN7). STN7-dependent phosphorylation of chloroplast proteins leads on the one hand to optimization of photosynthesis in response to changing light conditions (*via *the reversible reallocation of light-harvesting antennae called state transitions) and on the other hand to a retrograde signal (Bonardi et al., 2005; Rochaix, 2012). Another chloroplast protein kinase, CHLOROPLAST SENSOR KINASE (CSK), couples plastoquinone redox state to the regulation of chloroplast gene expression (Puthiyaveetil et al., 2012). The *soldat8 *mutation in the chloroplastic RNA polymerase* SIGMA SUBUNIT 6* (*SIG6*) gene increases the tolerance of seedlings to ^1^O_2_ (Coll et al., 2009), which links chloroplast transcriptional control to the ROS signaling. The RNA-binding chloroplast protein GENOMES UNCOUPLED 1 (GUN1) is implicated both in chloroplast translation and tetrapyrrole biosynthesis and is somehow involved in retrograde signaling (Czarnecki et al., 2011; Woodson et al., 2012). GUN1, and one of the key components of tetrapyrrole biosynthesis, the ChlH subunit of magnesium chelatase, are also involved in abscisic acid signaling (Shen et al., 2006; Koussevitzky et al., 2007; Cutler et al., 2010; Shang et al., 2010). The heme, the product of a side branch of tetrapyrrole biosynthesis, exits chloroplasts to be used as a cofactor by numerous hemoproteins in the cell and to provide positive feedback on transcription of nuclear genes that encode chlorophyll-binding proteins of chloroplasts (Nott et al., 2006; Woodson et al., 2011, 2012; Czarnecki and Grimm, 2012). The disturbance of the cell affects the delicate physiological equilibrium of the chloroplasts resulting in elevated ROS production.

## CHLOROPLASTIC ROS AS SIGNALS

Plant cells have over the course of evolution learned to use chloroplastic ROS for signaling purposes. Several studies have demonstrated a central role for the highly reactive singlet oxygen (^1^O_2_) as a chloroplastic signal involved in the regulation of plant cell death. PS II and its light-harvesting antennae produce ^1^O_2_ when light-excited chlorophylls adopt the rare triplet state and then reduce triplet oxygen (Krieger-Liszkay et al., 2008). Production of ^1^O_2_ is enhanced when the light-excited electrons cannot escape PS II chlorophylls because the downstream components of electron transfer chain (mainly the plastoquinone pool) are already over-reduced, a situation typical of excessive illumination. ^1^O_2_ readily reacts with lipids, proteins, and pigments and is rapidly quenched by water, which makes its diffusion distance from the site of production shortest among all ROS (Asada, 2006). For that reason ^1^O_2_ is unlikely to leave the chloroplasts, but several products of ^1^O_2_-dependent lipid or carotenoid oxidation, including oxylipins (op den Camp et al., 2003; Przybyla et al., 2008) and volatile β-cyclocitral (Ramel et al., 2012), are suspected to act as the ^1^O_2_-dependent retrograde signal.

The Arabidopsis* flu* mutant (Meskauskiene et al., 2001) has been used as a genetic tool to identify ^1^O_2_-responsive genes and to dissect signaling pathways triggered by ^1^O_2_ production in chloroplasts. The chloroplast-localized FLU protein inhibits one of the early enzymes of tetrapyrrole biosynthesis. *Flu *seedlings are unable to control the biosynthetic pathway through negative feedback and accumulate the chlorophyll precursor protochlorophyllide in the dark. Being transferred to light, the seedlings bleach and die due to the massive generation of ^1^O_2_ in their chloroplasts. This death is primarily caused by a profound reprogramming of nuclear transcription rather than by mere chemical toxicity of ^1^O_2_ (op den Camp et al., 2003). In addition, shortly after the exposure of *flu *seedlings to light, their chloroplasts rupture releasing the soluble stroma into the cytosol – this resembles the leakage of mitochondrial proteins to the cytosol during the mitochondria-triggered PCD. Two homologous chloroplast proteins EXECUTER1 and EXECUTER2 (**Figure 2**) conserved in higher plants are involved in this process, although their exact role is unknown (Wagner et al., 2004; Lee et al., 2007; Kim et al., 2012). It should be noted that although ^1^O_2_-dependent PCD is significantly exacerbated in *flu*, it is not confined to the mutant but is also observed in wild-type Arabidopsis under severe light stress (Kim et al., 2012). The sensory and signaling systems involved in the transmission of the chloroplastic ^1^O_2_-dependent signal to nucleus have not been identified, but it has been suggested that nuclear topoisomerase VI could act as an integrator of ^1^O_2_-dependent signal in regulating nuclear gene expression (Šimková et al., 2012).

Apart from triggering PCD, the transcriptional reprogramming of *flu* induces many genes of stress response and leads to rapid accumulation of SA, inducing a defense pathway characteristic of plant reaction to pathogens or wounding (Ochsenbein et al., 2006; Lee et al., 2007). One of the mechanisms triggering this pathway exploits the calcium-sensing protein CAS localized to chloroplast thylakoids (**Figure 2**). Regulation of CAS activity is linked to the state of photosynthetic electron transfer chain. CAS has earlier been shown to be involved in high light acclimation of the green alga *Chlamydomonas reinhardtii *(Petroutsos et al., 2011) and it is phosphorylated by the thylakoid protein kinase STN8 (Vainonen et al., 2008), a paralog of STN7, which suggests a link between the CAS activity and the redox state of the plastoquinone pool. However, CAS is not only involved in light-dependent retrograde signaling: also various abiotic or biotic stress stimuli activate CAS through a yet unknown mechanism. This activation leads to reallocation of Ca^2+^ ions within the chloroplast and to accumulation of ^1^O_2_, which, in turn, initiates defense responses through an unidentified retrograde signal (Nomura et al., 2012). Thus, CAS appears to act in the ^1^O_2_-dependent retrograde signaling pathway discussed above.

Another source of ROS in chloroplasts is PS I. Its electron-donor side generates ${\text{O}}_{2}^{•-}$ that is scavenged by chloroplast SOD to form H_2_O_2_ (Asada, 2006). H_2_O_2_, in turn, is reduced to water by a number of enzymes including ascorbate peroxidase (APX), peroxiredoxin, and glutathione peroxidase. H_2_O_2_ produced in chloroplasts gives rise to retrograde signals. The signaling is not well understood and might be a combination of passive diffusion of H_2_O_2_with indirect pathways including hormonal (abscisic acid) signaling (Mullineaux and Karpinski, 2002; Galvez-Valdivieso and Mullineaux, 2010). The possibility of H_2_O_2_ leakage from chloroplasts is supported by the fact that a knockout of cytosolic APX1 leads to hypersensitivity of the photosynthetic apparatus to light stress (Davletova et al., 2005). Diffusion of H_2_O_2_ from chloroplasts has also been demonstrated *in vitro *(Mubarakshina et al., 2010). Aquaporins in the chloroplast envelope (**Figure 2**) seem to be involved in this H_2_O_2_ leakage (Borisova et al., 2012), but how the aquaporins are regulated is unknown. In any case, H_2_O_2_ itself is not likely to be the retrograde signaling substance that directly affects nuclear gene expression. More probably, it is sensed by compartment-specific redox-sensitive components, which mediate the signal to the nucleus (Sierla et al., 2012). Oxidized proteins or peptides have been suggested as one of the possible downstream mediators of such H_2_O_2_ signaling (Wrzaczek et al., 2009; Møller and Sweetlove, 2010).

## THE FRUSTRATING COMPLEXITY OF ROS RESPONSES

One of the most frequently employed tools to investigate the role of ${\text{O}}_{2}^{•-}$ in the chloroplast is the herbicide methyl viologen (MV; also known as paraquat). MV accelerates the production of ${\text{O}}_{2}^{•-}$ by PS I and inhibits APX, leading to the accumulation of H_2_O_2_ in MV-treated plants (Mano et al., 2001). Comparison of the transcriptional responses to ^1^O_2_ and H_2_O_2_ using the *flu* mutant and the plants treated with MV demonstrated the specific and to a large extent antagonistic effect of these two chloroplastic ROS on gene expression (op den Camp et al., 2003; Gadjev et al., 2006; Laloi et al., 2007). Interestingly, the transcriptional response to apoplastic H_2_O_2_ produced during oxidative burst has little similarity to the effect of either chloroplastic ${\text{O}}_{2}^{•-}$/H_2_O_2_ or chloroplastic ^1^O_2_ (Gadjev et al., 2006; Miller et al., 2009; Petrov et al., 2012; Sierla et al., 2012). This illustrates a remarkable specificity of cellular responses to different types and subcellular sources of ROS production. This also demonstrates the complexity of ROS signaling and raises the question of the mechanisms responsible for such specificity.

Clustering results of microarray experiments involving ROS production in different subcellular compartments reveals distinct temporal signatures. For example, the gene expression profiles 4 h after elicitation with flg22 (a 22-amino acid fragment of the bacterial flagellar protein flagellin, which induces an apoplastic oxidative burst *via* the activation of NADPH oxidase) have similarities to the profiles induced by ozone, while 12 h after flg22 treatment the expression profile resembled that of chloroplastic ROS production induced by MV (Sierla et al., 2012). Ozone triggers generation of ROS in the apoplast, which leads to subsequent chloroplastic ROS production and transcriptional up-regulation of 25 (out of 44) Arabidopsis *CRK* genes, but the activation profile of these genes differs from that induced by high light (Wrzaczek et al., 2010). Thus, both temporal and spatial aspects appear to be involved in determining the specificity of ROS. In addition, the outcome is most likely dictated by the specific combinations of ROS (${\text{O}}_{2}^{•-}$, H_2_O_2_, or ^1^O_2_). It is unlikely that, for example, merely a change in the cytoplasmic redox state could carry the information about the subcellular source of H_2_O_2_. Therefore, as proposed (Møller and Sweetlove, 2010), the signal transduction would require specific and distinct sensory systems for the different ROS in diverse subcellular compartments.

The involvement of chloroplasts in plant systemic signaling has also started to emerge recently (Joo et al., 2005; Szechyñska-Hebda et al., 2010). Excessive illumination of Arabidopsis rosettes resulted in the propagation of an electric signal as measured by changes in plasma membrane potential of bundle sheath cells of leaf central veins. The signal was systemic, i.e., it also spread over the shaded leaves of the entire rosette. It correlated with transients of H_2_O_2_ concentration and was altered in the mutant deficient in cytosolic APX2. Besides, the signal was deregulated by the inhibitors of photosynthetic electron transfer and blocked by a Ca^2+^ channel inhibitor. These observations suggest that information on light conditions perceived by the chloroplast photosynthetic apparatus is communicated to the cell, most likely through ROS production, and then propagated along the plant in a Ca^2+^-channel dependent way (Szechyñska-Hebda et al., 2010). The possible integration of this pathway with a systemic NADPH oxidase-dependent signal (Miller et al., 2009) is the subject of further research.

The high focused localization of ROS signaling events raises the issue of organelle spatial organization inside the cell. Stromules, the transient protrusions of organellar surfaces that are known to be induced by stress, could be one of the mediators of this focused organellar cross-talk (Leister, 2012). Besides, all organelles move and dynamically associate with each other, and the role of this movement in stress response starts to be recognized (Suzuki et al., 2012). For example, the bacterial elicitor harpin leads to HR accompanied by redistribution of mesophyll cell chloroplasts in tobacco (Boccara et al., 2007). During the last 15 years the laws of organellar movement have started to be revealed, but the consequences of dynamic physical proximity and contact between the organelles are unknown (Suetsugu et al., 2010; Sakai and Haga, 2012). Recent studies demonstrate impairment of stress reactions in the Arabidopsis mutants which are unable to move chloroplasts in response to light stimuli or to dock them to the plasma membrane (Schmidt von Braun and Schleiff, 2008; Goh et al., 2009; Oliver et al., 2009; Lehmann et al., 2011).

## CONCLUDING REMARKS

Research performed over the last two decades has made it clear that ROS signaling connects events that take place in very different subcellular locations, most importantly (but not limited to) the apoplast, the chloroplast, and the nucleus. To achieve the elaborate and fine-tuned responses to biotic and abiotic stimuli that we observe on transcriptional, biochemical, and physiological level, intense and strictly controlled communication between the subcellular “crime scenes” must take place. While some components of this information exchange have been proposed, we still lack a thorough understanding on how the apoplast, the chloroplast, and the nucleus keep in touch. It will be perhaps one of the major challenges of ROS research in plants to understand ROS-induced signaling pathways between different organelles. Once we find out which components transmit information under specific conditions, we will be able to generate an integrated view of ROS signaling and its role in environmental adaptation.

## Conflict of Interest Statement

The authors declare that the research was conducted in the absence of any commercial or financial relationships that could be construed as a potential conflict of interest.

## Acknowledgments

Research in the Plant Stress group of Professor Jaakko Kangasjärvi is supported by Biocentrum Helsinki, the Academy of Finland, the University of Helsinki, and the ERA-PG.
